# Medial prefrontal area reductions, altered expressions of cholecystokinin, parvalbumin, and activating transcription factor 4 in the corticolimbic system, and altered emotional behavior in a progressive rat model of type 2 diabetes

**DOI:** 10.1371/journal.pone.0256655

**Published:** 2021-09-10

**Authors:** Ryosuke Ochi, Naoto Fujita, Natsuki Goto, Kaho Takaishi, Takaya Oshima, Son Tien Nguyen, Hisao Nishijo, Susumu Urakawa

**Affiliations:** 1 Department of Musculoskeletal Functional Research and Regeneration, Graduate School of Biomedical and Health Sciences, Hiroshima University, Minami-ku, Hiroshima, Japan; 2 System Emotional Science, Faculty of Medicine, University of Toyama, Sugitani, Toyama, Japan; Chiba Daigaku, JAPAN

## Abstract

Metabolic disorders are associated with a higher risk of psychiatric disorders. We previously reported that 20-week-old Otsuka Long-Evans Tokushima fatty (OLETF) rats, a model of progressive type 2 diabetes, showed increased anxiety-like behavior and regional area reductions and increased cholecystokinin-positive neurons in the corticolimbic system. However, in which stages of diabetes these alterations in OLETF rats occur remains unclear. We aimed to investigate anxiety-like behavior and its possible mechanisms at different stages of type 2 diabetes in OLETF rats. Eight- and 30-week-old OLETF rats were used as diabetic animal models at the prediabetic and progressive stages of type 2 diabetes respectively, and age-matched Long-Evans Tokushima Otsuka rats served as non-diabetic controls. In the open-field test, OLETF rats showed less locomotion in the center zone and longer latency to leave the center zone at 8 and 30 weeks old, respectively. The areas of the medial prefrontal cortex were smaller in the OLETF rats, regardless of age. The densities of cholecystokinin-positive neurons in OLETF rats were higher in the lateral and basolateral amygdala only at 8 weeks old and in the anterior cingulate and infralimbic cortices and hippocampal cornu ammonis area 3 at both ages. The densities of parvalbumin-positive neurons of OLETF rats were lower in the cornu ammonis area 2 at 8 weeks old and in the prelimbic and infralimbic cortices at both ages. No apoptotic cell death was detected in OLETF rats, but the percentage of neurons co-expressing activating transcription factor 4 and cholecystokinin and parvalbumin was higher in OLETF rats at both ages in the anterior cingulate cortex and basolateral amygdala, respectively. These results suggest that altered emotional behavior and related neurological changes in the corticolimbic system are already present in the prediabetic stage of OLETF rats.

## Introduction

Metabolic disorders such as obesity and type 2 diabetes can induce several psychiatric disorders. Patients with diabetes often have a high prevalence of cognitive impairment, depression, and anxiety [1,2]. These psychiatric disorders are also associated with obesity and metabolic syndrome [3–5]. Obesity and metabolic syndrome likely precede the onset of type 2 diabetes; therefore, psychiatric disorders could already be present in the early stages of type 2 diabetes. Indeed, prediabetic mice reportedly exhibit cognitive impairment and depressive-like behavior [6]. However, little has been reported on the comorbidity of anxiety in different stages of type 2 diabetes and the neural mechanisms. Individuals with anxiety at prediabetic stages are more likely to develop type 2 diabetes [7]. In addition, patients with type 2 diabetes and anxiety have a higher risk of mortality than non-diabetic and non-anxious individuals [8]. To effectively treat and prevent anxiety in patients with type 2 diabetes, it is important to understand its neural mechanisms.

Otsuka Long-Evans Tokushima fatty (OLETF) rats are a type 2 diabetes model developed by selective breeding of outbred Long-Evans rats [9]. OLETF rats chronically develop type 2 diabetes with age. We previously confirmed that OLETF rats showed a progressive course of type 2 diabetes: mild hyperglycemia and normal plasma insulin level at 8 weeks old, indicating the prediabetic stage; hyperglycemia and hyperinsulinemia at 20 weeks old, indicating the early stage of type 2 diabetes; and severe hyperglycemia and a mixture of hyperinsulinemia and hypoinsulinemia at 30 weeks old, indicating the progressive stage of type 2 diabetes from non-insulin-dependent diabetes mellitus to insulin-dependent diabetes mellitus [9,10]. We recently reported that 20-week-old OLETF rats exhibited increased anxiety-like behavior in the open-field test, area reduction, and increased cholecystokinin (CCK)-positive neurons in the corticolimbic system [11]. Previous results obtained in OLETF rats between 10 and 20 weeks old also showed increased anxiety-like behaviors in the elevated plus maze, light–dark box, and open-field tests [12–14]. However, anxiety-like behavior remains to be compared in the prediabetic and progressive stages of type 2 diabetes, namely, OLETF rats of different ages.

Brain region shrinkage is reportedly associated with anxiety in patients with type 2 diabetes. Gray matter volume was reduced in patients with type 2 diabetes with anxiety and depression compared to patients with type 2 diabetes without anxiety or depression [15]. Furthermore, patients with obsessive–compulsive disorder and generalized anxiety disorder showed decreased gray and white matter volume in emotion-related regions, including the medial prefrontal cortex (mPFC), which is associated with anxiety severity [16]. Importantly, we also demonstrated that 20-week-old OLETF rats in the early stage of type 2 diabetes exhibited lower brain weight as well as area reductions in the corticolimbic system, including the mPFC [11]. Therefore, shrinkage of specific regions in the corticolimbic system could be one of the neuroanatomical mechanisms underlying anxiety in type 2 diabetes. However, whether similar alterations are observed in OLETF rats in the prediabetic and progressive stages at different ages remains unclear.

CCK is a neurotransmitter peptide in the central nervous system [17] and is associated with anxiety. Systemic chemogenetic activation of CCK-positive inhibitory neurons reportedly increased anxiety-like behavior in the elevated plus maze test [18]. Furthermore, knockdown of CCK in the basolateral amygdala (BLA) decreased anxiety-like behavior in the elevated plus maze test [19]. Two types of CCK receptors have been identified: CCK-1 and CCK-2. OLETF rats lack the CCK-1 receptor due to a congenital genetic abnormality [20]. It has been suggested that OLETF rats exhibit increased anxiety-like behavior because of type 2 diabetes and/or deficits in CCK-1 receptor activity [12–14]. However, CCK injections in the mPFC, amygdala, hippocampus, and cerebral ventricle reportedly increased anxiety-like behavior via the activation of the CCK-2 receptor [21–25], whereas knockout of CCK-1 receptor did not generate anxiety-like behavior [26]. Therefore, the deficit in CCK-1 receptor alone in OLETF rats cannot sufficiently explain the increased anxiety-like behavior. Interestingly, we found that 20-week-old OLETF rats exhibited increased numbers of CCK-positive neurons in the corticolimbic system, including the mPFC, amygdala, and hippocampus, which might be indicative of the neural mechanisms causing increased anxiety-like behavior [11]. However, whether more CCK-positive neurons are present in the corticolimbic system in OLETF rats in the prediabetic and progressive stages of type 2 diabetes has not been clarified.

Basket cells in the mPFC, amygdala, and hippocampus are neurochemically classified into two types: CCK- or parvalbumin (PV)-positive neurons [27–29]. PV-positive neurons in these regions are also reportedly involved in anxiety-like behavior [30–32]. Contrary to CCK-positive neurons, chemogenetic inhibition of PV-positive neurons in the BLA increased anxiety-like behavior in the open-field and elevated plus maze tests [31]. CCK- and PV-positive neurons are divided into large or small types according to the size of the somata, especially in the amygdala [11,33–35]. Although functional differences between large and small types of CCK-positive neurons have not been well defined, the neurochemical properties of each type are reportedly different: large CCK-positive neurons co-express calbindin and neurokinin 1 receptor, whereas small CCK-positive neurons co-express calretinin and vasoactive intestinal polypeptide [33,34]. In addition, although functional differences between large and small types of PV-positive neurons are not fully understood, we previously reported that an increased number of small PV-positive neurons in the BLA, achieved through rearing in an enriched environment, was associated with reduced anxiety-like behavior [35]. These results led us to hypothesize that anxiety-like behavior in OLETF rats could be associated with changes in the number of PV-positive cells in the corticolimbic system and be differentially associated with large and small types of CCK- and PV-positive neurons in the amygdala.

Spatial memory impairment in streptozotocin-induced diabetic mice reportedly involves neuronal apoptosis and the endoplasmic reticulum stress pathway in the hippocampus [36]. Endoplasmic reticulum stress induces upregulation of activating transcription factor 4 (ATF4), C/EBP-homologous protein (CHOP), and caspase-3, leading to neuronal apoptosis [37,38]. In contrast to streptozotocin-induced diabetic mice, Goto–Kakizaki (GK) rats, a rat model of type 2 diabetes, show apoptosis levels comparable to those of control Wistar rats, as demonstrated by caspase-3 and CHOP expression measurements and terminal deoxynucleotidyl transferase dUTP nick end labeling (TUNEL) staining [39,40]. These rats also showed increased ATF4 expression in PV-positive neurons in the hippocampus [39]. ATF4 is a transcriptional regulator involved in apoptosis that induces cholinergic neuron-specific losses, rather than global neuronal death [41]. Based on these results, we hypothesized that induction of apoptosis and/or alteration of ATF4 activity might be involved in the changes in the brains of OLETF rats at different stages of type 2 diabetes.

Collectively, the purpose of the present study was to clarify whether OLETF rats at the prediabetic and progressive stages of type 2 diabetes show increased anxiety-like behavior, reduced brain areas, and altered numbers of CCK- and PV-positive neurons in the corticolimbic system. Therefore, we investigated these factors at the prediabetic and progressive stages of type 2 diabetes, specifically at 8 and 30 weeks old, respectively. To examine the possible mechanisms of area reduction and altered expression in CCK- and PV-positive neurons in OLETF rats, we explored the expression of caspase-3 and ATF4. The results of the present study suggest that OLETF rats at the prediabetic stage already exhibit altered emotional behavior, associated area reduction, and altered numbers of CCK- and PV-positive neurons in the corticolimbic region.

## Materials and methods

### Animals

**Fig 1:**
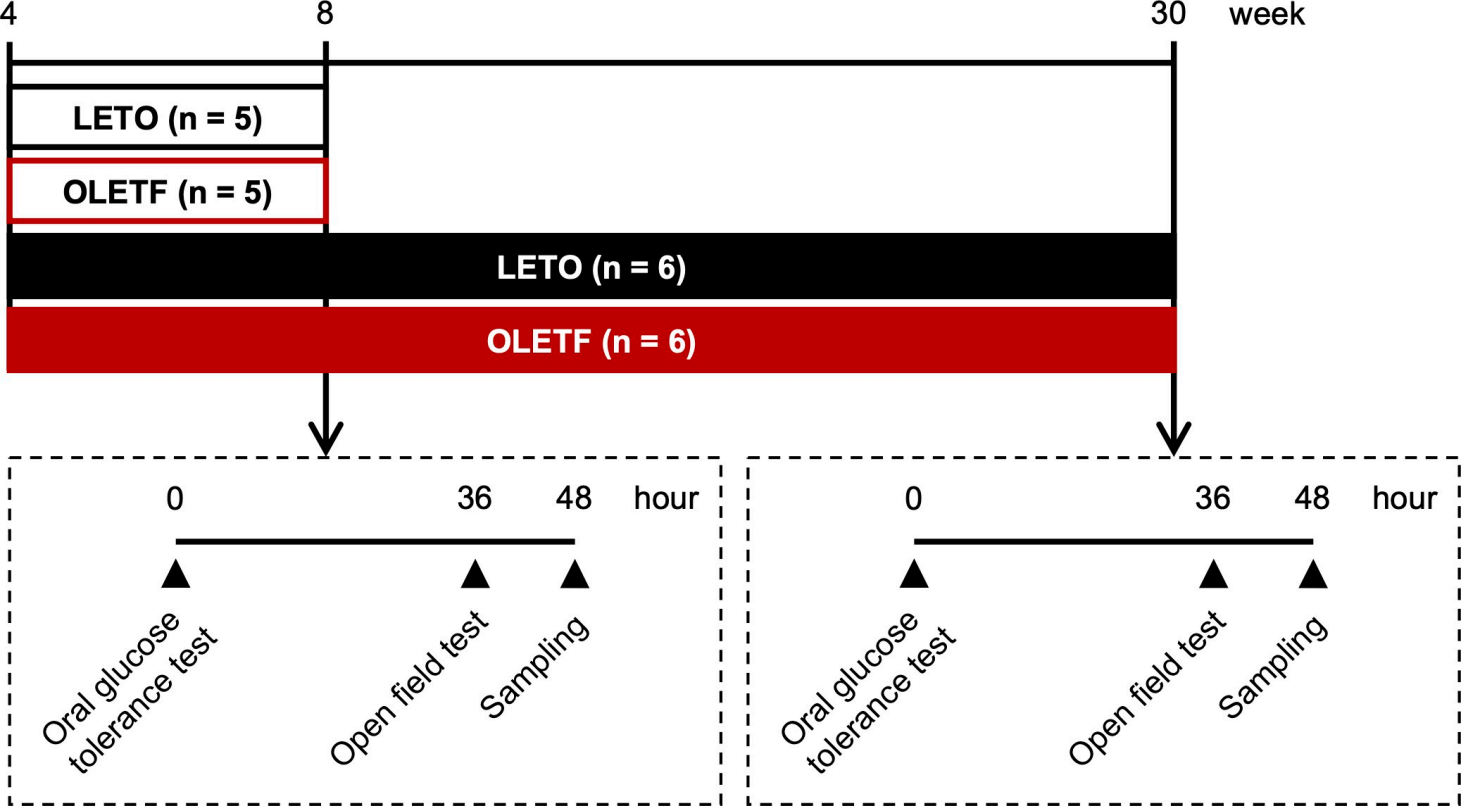
Experimental timeline. Male LETO and OLETF rats were housed and given free access to food and water. At the age of 8 or 30 weeks, an oral glucose tolerance test was conducted. After 36 h, an open-field test was performed. At 12 h after the open-field test, the brain samples were collected.

All experiments were conducted in accordance with the Hiroshima University Regulations for Animal Experimentation and National Institute of Health Guidelines for the Care and Use of Laboratory Animals and were approved by the Institutional Animal Care and Use Committee of Hiroshima University (A16-5). Four-week-old male OLETF and control Long-Evans Tokushima Otsuka (LETO) rats were purchased from Hoshino Laboratories Animals (Ibaraki, Japan). The rats were divided into four groups according to age and strain: 8-week-old LETO rats (n = 5), 8-week-old OLETF rats (n = 5), 30-week-old LETO rats (n = 6), and 30-week-old OLETF rats (n = 6) (Fig 1). The rats were housed in cages (three rats per cage) under a 12-h light–dark cycle (lights on from 8:00 to 20:00) and controlled temperature (22°C ± 2°C). The rats were given free access to food and water. Body weight and food intake were measured weekly. We used the same rats than in our previous study [10].

A traumatic brain injury was induced in a 9-week-old male Sprague-Dawley rat for use as a positive control for immunohistochemistry for caspase-3 [42]. The rat was deeply anesthetized with isoflurane and sodium pentobarbital. A craniotomy was made over the right parietal cortex and injury was produced using a 25-gauge needle. The rat was then returned to the home cage.

### Oral glucose tolerance test and plasma insulin measurement

At the age of 8 or 30 weeks, an oral glucose tolerance test was performed to determine glucose tolerance relative to body weight, as described previously [43]. The rats fasted for 12 h to deplete glycogen stores in the liver for metabolic analysis for another study [43]. Blood samples were collected from the lateral caudal vein before and at 30, 60, and 120 min after glucose administration. Whole-blood glucose concentration was measured using an amperometric quinoprotein glucose dehydrogenase electrode method (ACCU-CHEK ST meter; Roche, Basel, Switzerland). The blood samples were centrifuged at 3000 rpm for 10 min at room temperature, and the plasma was stored at -80°C until further analysis. Plasma insulin concentration was measured using an enzyme-linked immunosorbent assay kit (M1101; Morinaga, Yokohama, Japan) according to the manufacturer’s guidelines. The integrated values of glucose and insulin levels during the test and the area under the curves (AUCs) were calculated using the trapezoidal method.

### Open-field test

At 36 h after the oral glucose tolerance test, an open-field test was conducted during the dark phase, as described in our previous study [11]. The rats fasted during and after the test. At the beginning of the test, each rat was placed in the center zone (45 cm in diameter) of a circular field (90 cm in diameter) surrounded by a wall (70 cm in height). The illumination level in the central zone was 381 lx. Each rat was allowed to freely explore the field for 10 min and was then placed back in its home cage. After each test, the field was cleaned with 70% ethanol to remove any odors. Each test was recorded using an overhead digital camera (EX-ZR1000; Casio, Tokyo, Japan). Locomotion and time in the center zone were analyzed using AnimalTracker (http://animaltracker.elte.hu [44]), and rearing frequency (rising on the hind limbs), duration of grooming, inactivity (absence of movement of all limbs), latency to leave the center zone (first placement of all four paws into the peripheral zone), and number of re-entrances to the center zone (all four paws placed into the center zone after first escape from this zone) were manually analyzed using ImageJ software (National Institutes of Health, Bethesda, MD, USA). Velocities of locomotive behaviors were calculated as locomotion divided by durations of locomotive behaviors except for grooming and inactivity in the center and peripheral zones.

### Tissue preparation

Twelve hours after the open-field test, the LETO and OLETF rats were sacrificed by an overdose of sodium pentobarbital. Twenty-four hours after traumatic brain injury, the Sprague-Dawley rat was sacrificed by an overdose of sodium pentobarbital and perfused transcardially with 4% paraformaldehyde in 0.1 M phosphate buffer. Body weight was measured immediately before sacrifice. The brains were removed, weighed, and fixed in 4% paraformaldehyde in 0.1 M phosphate buffer for 24 h. After fixation, the brains were placed in 20% sucrose until they sank and were coronally sectioned (30 μm in thickness) using a cryostat. Sections were transferred into a cryoprotectant solution at -30°C until further use. Six serial sections were processed for one staining each: one for CCK, one for PV, one for caspase-3, and three for immunofluorescence of ATF4 with NeuN, CCK, and PV.

### Immunohistochemistry

Immunoperoxidase staining procedures were similar to those in our previous study [11]. Free-floating sections were rinsed three times in phosphate-buffered saline (PBS) or PBS containing 0.25% Triton X-100 (PBS-T) between each incubation step. The sections were quenched for 10 min in 2% H_2_O_2_/20% methanol in PBS, processed for antigen retrieval using citrate buffer for staining for CCK and caspase-3, and blocked using 3% normal horse or goat serum in PBS-T for 30 min at room temperature. After rinsing, the sections were incubated with rabbit anti-CCK antibody (1:10,000; Sigma, St. Louis, MO, USA), mouse anti-PV antibody (1:10,000; Sigma), or mouse anti-pro and active caspase-3 antibody (1:500; Novus Biologicals, Centennial, CO, USA) in 1% blocking solution at 4°C overnight. Thereafter, the sections were incubated with biotinylated anti-rabbit or anti-mouse antibody (1:500; Vector Laboratories, Burlingame, CA, USA) on ice for 1 h, incubated with avidin–biotin–peroxidase complex (ABC-Elite; Vector Laboratories), and reacted with diaminobenzidine. Finally, the sections were mounted on gelatinized glass slides, dehydrated in ethanol, cleared in xylene, and cover-slipped with Entellan New (Merck, Darmstadt, Germany).

For immunofluorescence, free-floating sections were rinsed three times in PBS or PBS-T between each incubation step. The sections were processed for antigen retrieval using citrate buffer and blocked with 3% normal goat serum in PBS-T for 30 min at room temperature. After rinsing, the sections were incubated simultaneously with mouse anti-ATF4 antibody (1:1000; Novus Biologicals) and rabbit anti-NeuN antibody (1:4000; Merck Millipore, Burlington, MA, USA), rabbit anti-CCK antibody (1:4000; Sigma), or rabbit anti-PV antibody (1:5000; Swant, Marly, Switzerland) at 4°C. Two days later, the sections were incubated with biotinylated anti-mouse antibody (1:500; Vector Laboratories) on ice for 2 h, an Alexa Fluor 555–conjugated anti-rabbit antibody (1:500; Cell Signaling Technology, Danvers, MA, USA) on ice for 1 h, and a fluorescein avidin D (1:500; Vector Laboratories) on ice for 1 h. Finally, the sections were mounted on MAS-coated glass slides with mounting medium containing 4′,6-diamino-2-phenylindole (DAPI; for staining of nuclei) (Vector Laboratories). Negative control sections were produced by omitting each primary antibody, and no non-specific labeling of cells was observed in any of the control sections.

### Measurement of brain areas

Brain areas in the corticolimbic regions were measured as described in our previous study [11]. Slides were coded until the analysis was completed. Images of brain regions were obtained using a light microscope (BX51; Olympus, Tokyo, Japan) with a 2× dry objective lens equipped with a digital camera (DP70; Olympus) and analyzed using ImageJ software. Data were collected from both hemispheres and averaged.

The anatomical locations of the mPFC, basolateral amygdaloid complex (BLC), and hippocampus were determined using PV-stained sections with reference to the atlas [45]. We measured the areas of the mPFC including the anterior cingulate cortex (ACC), prelimbic cortex (PL), and infralimbic cortex (IL) in three sections at anterior to posterior (AP) 3.12, 2.94, and 2.76 mm from the bregma; BLC including the lateral amygdala (LA) and BLA in four sections at AP -1.92, -2.10, -2.28, and -2.46 mm from the bregma; hippocampus including the hippocampal cornu ammonis (CA) area 1, CA2, CA3; and dentate gyrus (DG) in two sections at AP -2.98 and -3.16 mm from the bregma.

### Cell counting

The number of CCK- and PV-positive neurons was counted in the same regions used for the measurements of brain areas using immunoperoxidase-stained sections as described in our previous study [11]. The analysis was performed using a light microscope (BX51) with a 20× dry objective lens. A neuron with CCK and PV immunoreactivity above the background level was considered as a CCK- and PV-positive neuron. In the BLC, CCK- and PV-positive neurons were classified into large and small types using the following criteria based on previous studies: large CCK-positive neurons (soma diameter >10 μm) and small CCK-positive neurons (soma diameter ≤10 μm) [11,33,34]; large PV-positive neurons (rectangular diameter >25 μm) and small PV-positive neurons (rectangular diameter ≤25 μm) [11,35]. Estimations of neuronal density (neurons/mm^2^) were calculated from both hemispheres and averaged. Slides were coded until the analysis was completed.

To determine whether NeuN-, CCK-, and PV-positive neurons co-express ATF4, immunofluorescent images were obtained using a confocal laser scanning microscope (FV1000-D; Olympus) with a 20× dry objective lens, converted to TIFF, and analyzed using ImageJ software. Two or three sections in each region from each brain were randomly scanned by obtaining 10 optical stacks of 1-μm thickness, and all positive neurons in each z-stack image were analyzed. The percentages of NeuN-, CCK-, and PV-positive neurons showing ATF4 co-expression were calculated in the same regions used for the measurement of brain areas. Only CCK- and PV-immunofluorescent-positive neurons with DAPI were counted. Slides and images were coded until the analysis was completed.

### Statistical analysis

Data are expressed as the mean ± standard error of the mean. All statistical analyses were performed using SPSS Statistics version 19.0 (IBM Japan, Tokyo, Japan). Differences between strain and age were evaluated using two-way analysis of variance followed by simple main effects analyses with Bonferroni adjustment. Correlations were evaluated using Spearman’s rank correlation coefficient with Bonferroni adjustment. Statistical significance was set at *p* < 0.05.

## Results

### Metabolic characteristics of OLETF rats

**Fig 2:**
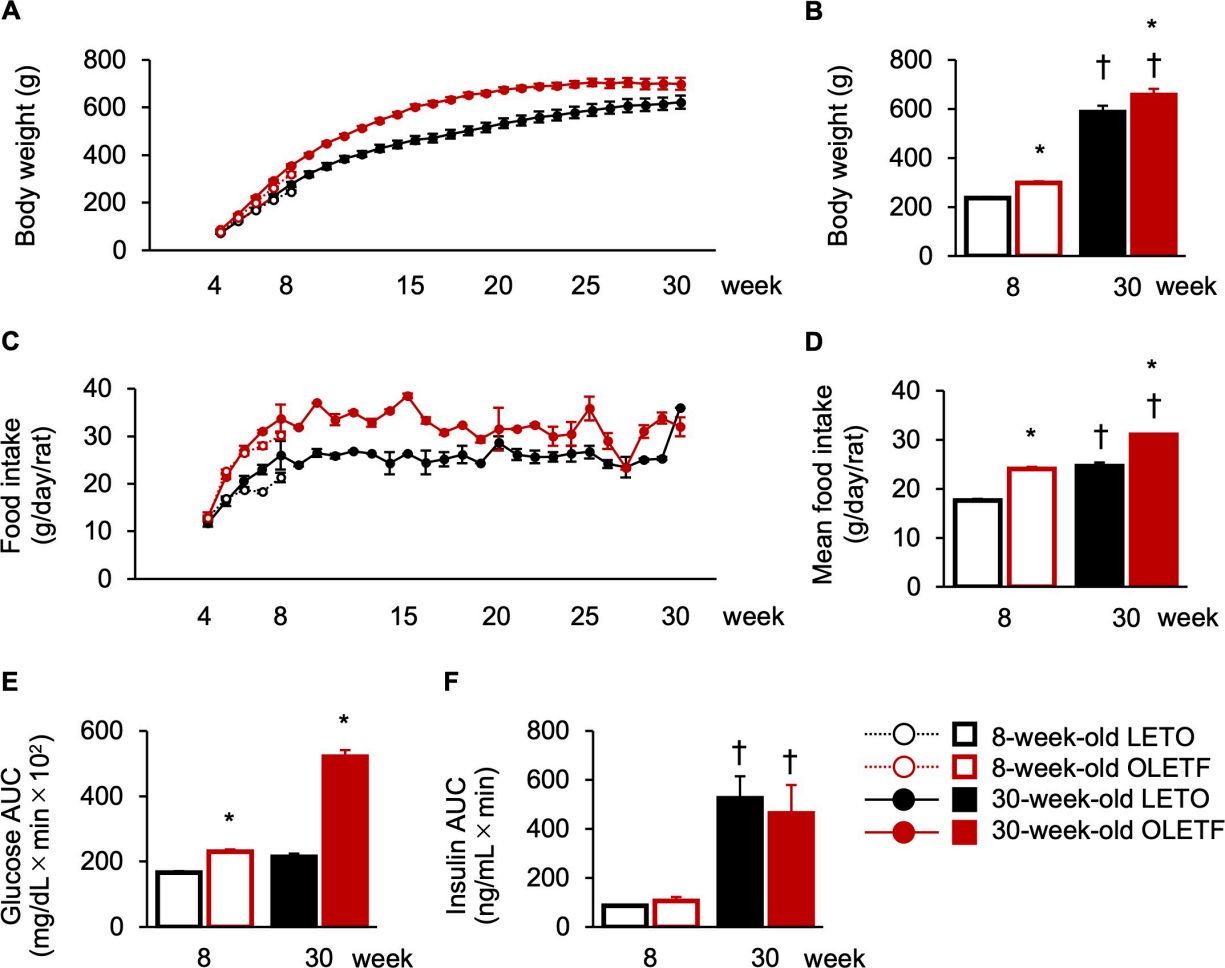
Pathological values. Body weights from 4 to 30 weeks of age (**A**) and just before euthanasia (**B**). Food intakes from 4 to 30 weeks of age (**C**) and mean food intakes of each experimental period (**D**). Area under the curves (AUCs) of glucose (**E**) and insulin (**F**) in the oral glucose tolerance test. Values represent mean ± standard error of means. * Significant difference from age-matched LETO rats, *p* < 0.01; † significant difference from same strain at 8 weeks of age, *p* < 0.01, two-way ANOVA.

To identify the pathological stages of type 2 diabetes in OLETF rats, we measured body weight, food intake, blood glucose, and plasma insulin levels. LETO rats continued to increase their body weights during the experimental period, whereas OLETF rats increased their body weights by around 25 weeks of age, but five of six 30-week-old OLETF rats showed a decrease in body weight at around 25 weeks old (Fig 2A). However, food intakes were higher in OLETF rats than in LETO rats during the experimental period (Fig 2C). The body weight just before euthanasia of OLETF rats was significantly higher than that of 8- and 30-week-old LETO rats (Fig 2B), and there was a significant main effect of strain (F[1, 18] = 10.45, *p* < 0.01); meanwhile, the body weights of LETO and OLETF rats increased with age, and there was a significant main effect of age (F[1, 18] = 302.46, *p* < 0.01), but there was no significant interaction between strain and age. The mean food intakes during the experimental period were significantly higher in OLETF rats than those in LETO rats at both ages (Fig 2D), and there was a significant main effect of strain (F[1, 4] = 185.39, *p* < 0.01); meanwhile, the mean food intakes of LETO and OLETF rats increased with age, and there was a significant main effect of age (F[1, 4] = 220.17, *p* < 0.01), but there was no significant interaction between strain and age. The glucose AUCs of OLETF rats were significantly higher at 8 and 30 weeks old and dramatically increased with age; meanwhile, those of LETO rats also increased with age (Fig 2E), and there was a significant interaction between strain and age (F[1, 18] = 87.18, *p* < 0.01), significant simple main effects of strain (8 weeks old: F[1, 18] = 10.72, *p* < 0.01; 30 weeks old: F[1, 18] = 304.01, *p* < 0.01), and a significant simple main effect of age (LETO rats: F[1,18] = 6.83, *p* < 0.05; OLETF rats: F[1, 18] = 250.18, *p* < 0.01). The insulin AUCs were comparable between LETO and OLETF rats at both ages (Fig 2F); there was no significant main effect of strain, although insulin level was increased with age in both LETO and OLETF rats, and there was a significant main effect of age (F[1, 18] = 23.90, *p* < 0.01). There were two types in 30-week-old OLETF rats according to insulin level: two of six 30-week-old OLETF rats exhibited higher insulin and lower glucose AUCs (insulin: 601.38 ± 116.36; glucose: 49,312.50 ± 1501.78); four of six 30-week-old OLETF rats exhibited lower insulin and higher glucose AUCs (insulin: 187.73 ± 76.65; glucose: 57,742.50 ± 202.50). These results indicate that OLETF rats started shifting hypoinsulinemia from hyperinsulinemia at 30 weeks old; therefore, 30-week-old OLETF rats showed a decrease in body weight although their food intakes continued to be higher than those of LETO rats after 25 weeks old, as described in our previous report [10]. Taken together, the 8-week-old OLETF rats were in the prediabetic stage, as demonstrated by mild hyperglycemia, whereas the 30-week-old OLETF rats were in the progressive stage of type 2 diabetes, as demonstrated by severe hyperglycemia and a mixture of hyperinsulinemia and hypoinsulinemia.

### Open-field test

**Fig 3:**
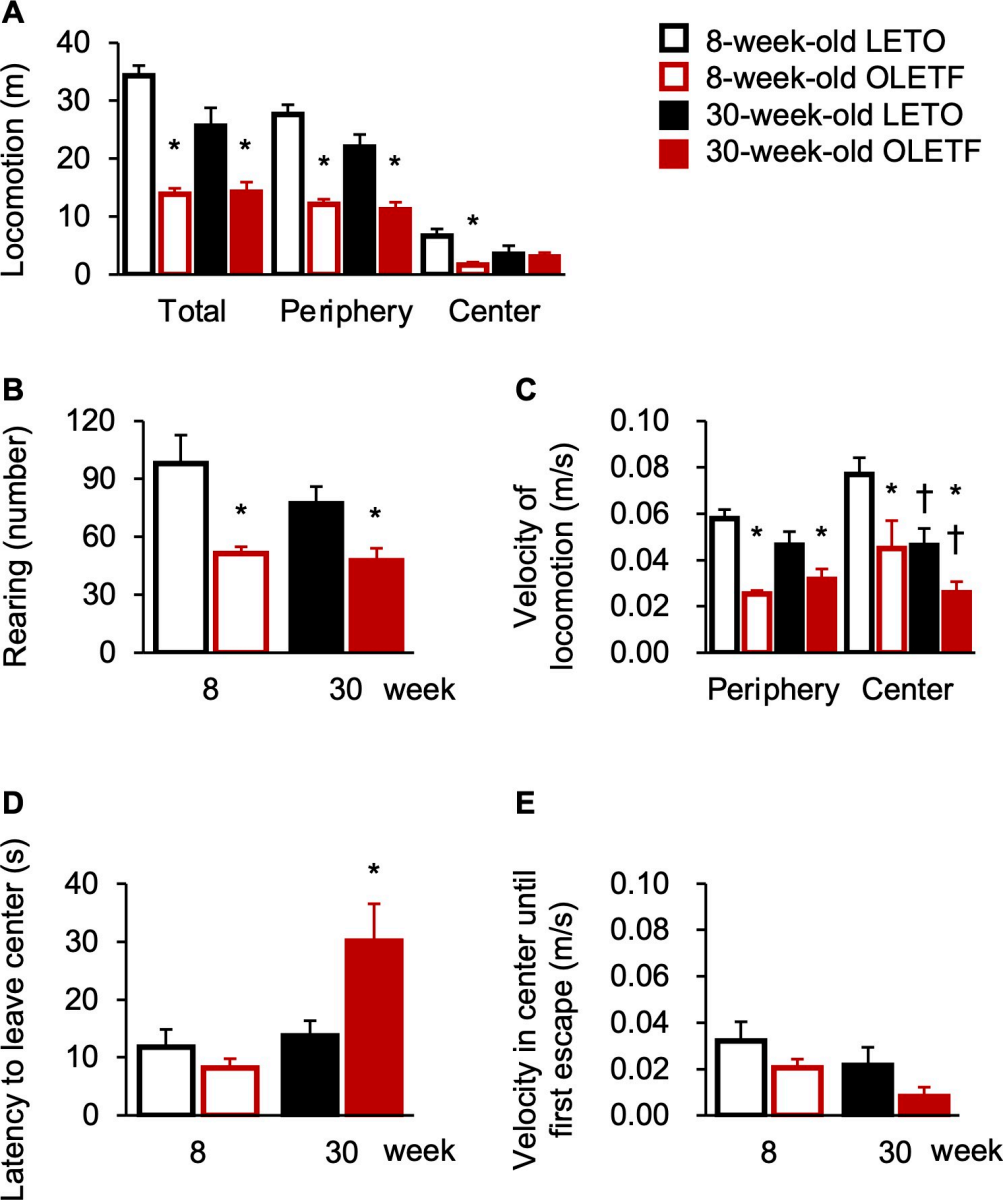
Behavioral assessment in the open field test. Locomotion in each zone (**A**) and number of rearing (**B**). Velocity of locomotive behaviors in each zone (**C**). Latency to leave the center zone (**D**) and velocity in the center zone until first escape (**E**). Values represent mean ± standard error of means. * Significant difference from age-matched LETO rats, *p* < 0.01, two-way ANOVA.

The locomotion of OLETF rats in the total and peripheral zones was significantly lower than that of LETO rats regardless of age (Fig 3A), and there was a significant main effect of strain (total zone: F[1, 18] = 51.76, *p* < 0.01; peripheral zone: F[1, 18] = 65.64, *p* < 0.01), but there was no significant main effect of age or interaction. The locomotion of OLETF rats in the center zone was significantly lower than that of rats only at 8 weeks old (Fig 3A), and there was a significant interaction between strain and age (F[1, 18] = 4.49, *p* < 0.05) and a significant simple main effect of strain at 8 weeks old (F[1, 18] = 10.16, *p* < 0.01), but not at 30 weeks old. Additionally, the rearing of OLETF rats was significantly less than that of LETO rats regardless of age (Fig 3B), and there was a significant main effect of strain (F[1, 18] = 15.85, *p* < 0.01), but there was no significant main effect of age or interaction. Based on these results, OLETF rats exhibited fewer locomotive behaviors; therefore, we analyzed non-locomotive behaviors, grooming, and inactivity. Indeed, OLETF rats showed hypolocomotive behaviors at each age. The duration of grooming in OLETF rats was significantly longer than that of LETO rats only at 8 weeks old (Table 1), and there was a significant interaction between strain and age (F[1, 18] = 5.78, *p* < 0.05) and a significant simple main effect of strain at 8 weeks old (F[1, 18] = 11.44, *p* < 0.01), but not at 30 weeks old. The duration of inactivity in OLETF rats was significantly longer than that in LETO rats only at 30 weeks old (Table 1); there was a significant interaction between strain and age (F[1, 18] = 9.65, *p* < 0.01) and a significant simple main effect of strain at 30 weeks old (F[1, 18] = 19.15, *p* < 0.01), but not at 8 weeks old. The velocities of locomotive behaviors in the peripheral and center zones were lower in OLETF rats than in LETO rats regardless of age (Fig 3C), and there were significant main effects of strain (peripheral zone: F[1, 18] = 27.61, *p* < 0.01; center zone: F[1,18] = 10.81, *p* < 0.01); in addition, the velocity of locomotive behaviors in the center zone of LETO and OLETF rats decreased with age (F[1, 18] = 9.76, *p* < 0.01), but there was no significant interaction between strain and age. Next, we analyzed additional behavioral parameters in the center zone that were indicative of anxiety-related behaviors. Contrary to locomotion in the center zone, the latency to leave the center zone of OLETF rats was significantly longer than that of LETO rats only at 30 weeks old (Fig 3D), and there was a significant interaction between strain and age (F[1, 18] = 5.78, *p* < 0.05) and a significant simple main effect of strain at 30 weeks old (F[1, 18] = 8.56, *p* < 0.01), but not at 8 weeks old. Unlike the velocity of locomotive behaviors in the center zone during the experiment, the velocity of locomotive behaviors in the center zone until first escape was comparable between LETO and OLETF rats regardless of age (Fig 3E), and there was no significant main effect of strain and age. However, the time in the center zone and that after re-entering this zone were comparable between LETO and OLETF rats at both ages (Table 1); there was no significant main effect of strain or age. Furthermore, the number of re-entrances to the center zone was comparable between LETO and OLETF rats at both ages (Table 1); there was no significant main effect of strain and age. Similar to the locomotion in the center zone, the locomotion in the center zone without first escape trajectory was significantly lower in OLETF rats than that in LETO rats only at 8 weeks old (Table 1), and there was a significant interaction between strain and age (F[1, 18] = 4.65, *p* < 0.05) and a significant simple main effect of strain at 8 weeks old (F[1, 18] = 10.03, *p* < 0.01), but not at 30 weeks old.

**Table 1:** Behavioral assessments in the open field test.

	8 weeks of age		30 weeks of age	
	LETO	OLETF	LETO	OLETF
Grooming (s)	16.03 ± 2.40	68.66 ± 14.32 *	22.91 ± 6.73	24.91 ± 13.32
Inactivity (s)	17.91 ± 7.51	12.81 ± 5.48	26.14 ± 11.63	122.18 ± 25.96 *
Time in center (s)	86.97 ± 12.18	45.76 ± 16.79	71.43 ± 23.04	148.23 ± 45.05
Time in center after re-entering this zone (s)	75.19 ± 12.41	37.58 ± 16.42	57.70 ± 22.14	118.14 ± 48.38
Re-entrances to center (number)	9.20 ± 0.49	3.80 ± 1.07	6.33 ± 2.16	6.33 ± 1.43
Locomotion in center without first escape trajectory (m)	6.33 ± 1.15	1.54 ± 0.46 *	3.27 ± 1.39	2.89 ± 0.71

Values represent mean ± standard error of means.

* Significant difference from age-matched LETO rats, *p* < 0.05, two-way ANOVA.

### Brain weight and areas in the corticolimbic regions

**Fig 4:**
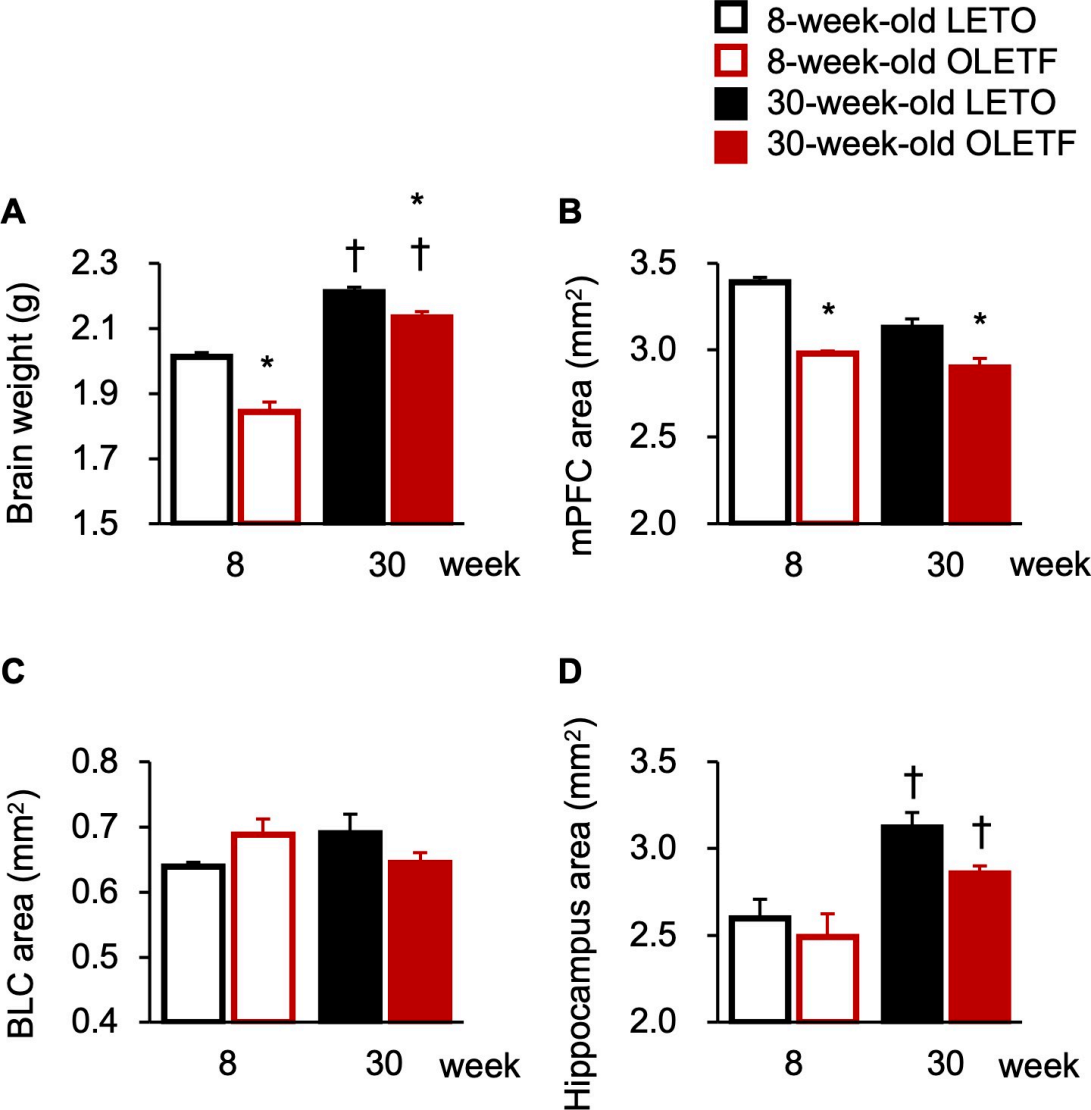
Brain weights and areas in the corticolimbic regions. Brain weights (**A**) and areas of the medial prefrontal cortex (mPFC; **B**), basolateral amygdaloid complex (BLC; **C**), and hippocampus (**C**). Values represent mean ± standard error of means. * Significant difference from age-matched LETO rats, *p* < 0.05; † significant difference from same strain at 8 weeks of age, *p* < 0.01, two-way ANOVA.

To examine the neuroanatomical mechanisms of anxiety-like phenotypes in OLETF rats, we measured the overall brain weight and quantified specific areas in the corticolimbic system. The brain weights of OLETF rats were significantly lower than those of LETO rats regardless of age (Fig 4A), and there was a significant interaction between strain and age (F[1, 18] = 5.29, *p* < 0.05), significant simple main effects of strain at both ages (8 weeks old: F[1, 18] = 32.86, *p* < 0.01; 30 weeks old: F[1, 18] = 8.23, *p* < 0.05), and significant simple main effects of age in both strains (LETO rats: F[1, 18] = 48.99, *p* < 0.01; OLETF rats: F[1, 18] = 105.10, *p* < 0.01). Furthermore, the brain weights increased with age regardless of strain (Fig 4A), and there were significant simple main effects of age in LETO and OLETF rats (LETO rats: F[1, 18] = 48.99, *p* < 0.01; OLETF rats: F[1, 18] = 105.10, *p* < 0.01). Next, we quantified the areas of specific brain regions in the corticolimbic system to determine whether these regions became smaller in OLETF rats. In line with the brain weights, the areas of the mPFC of OLETF rats were significantly smaller than those of LETO rats regardless of age (Fig 4B); there was a significant interaction between strain and age (F[1, 18] = 4.50, *p* < 0.05) and a significant simple main effect of strain at both ages (8 weeks old: F[1,18] = 41.61, *p* < 0.01; 30 weeks old: F[1, 18] = 15.36, *p* < 0.01). However, the areas of the BLC and hippocampus were comparable between LETO and OLETF rats at both ages (Fig 4C and 4D), and there was a significant interaction between strain and age in the BLC (F[1, 18] = 4.76, *p* < 0.05), but there were no significant simple main effects of strain at either age or significant main effect of strain or interaction in the hippocampus. In contrast, in line with the increased overall brain weights, the areas of the hippocampus were increased with age regardless of strain (Fig 4D); there was a significant main effect of age (F[1, 18] = 22.13, *p* < 0.01).

### CCK- and PV-positive neurons in the corticolimbic regions

To examine the additional mechanisms underlying anxiety-like phenotypes in OLETF rats, we measured the densities of CCK- and PV-positive neurons in the corticolimbic system. CCK- and PV-positive neurons in the LA and BLA consisted of different types according to somata sizes, as in previous studies [11,33–35]. We also classified CCK- and PV-positive neurons into large and small types based on somata diameters; however, alterations in large and small CCK- and PV-positive neurons were comparable between strains and ages. Therefore, we combined the densities of the large and small neurons.

**Fig 5:**
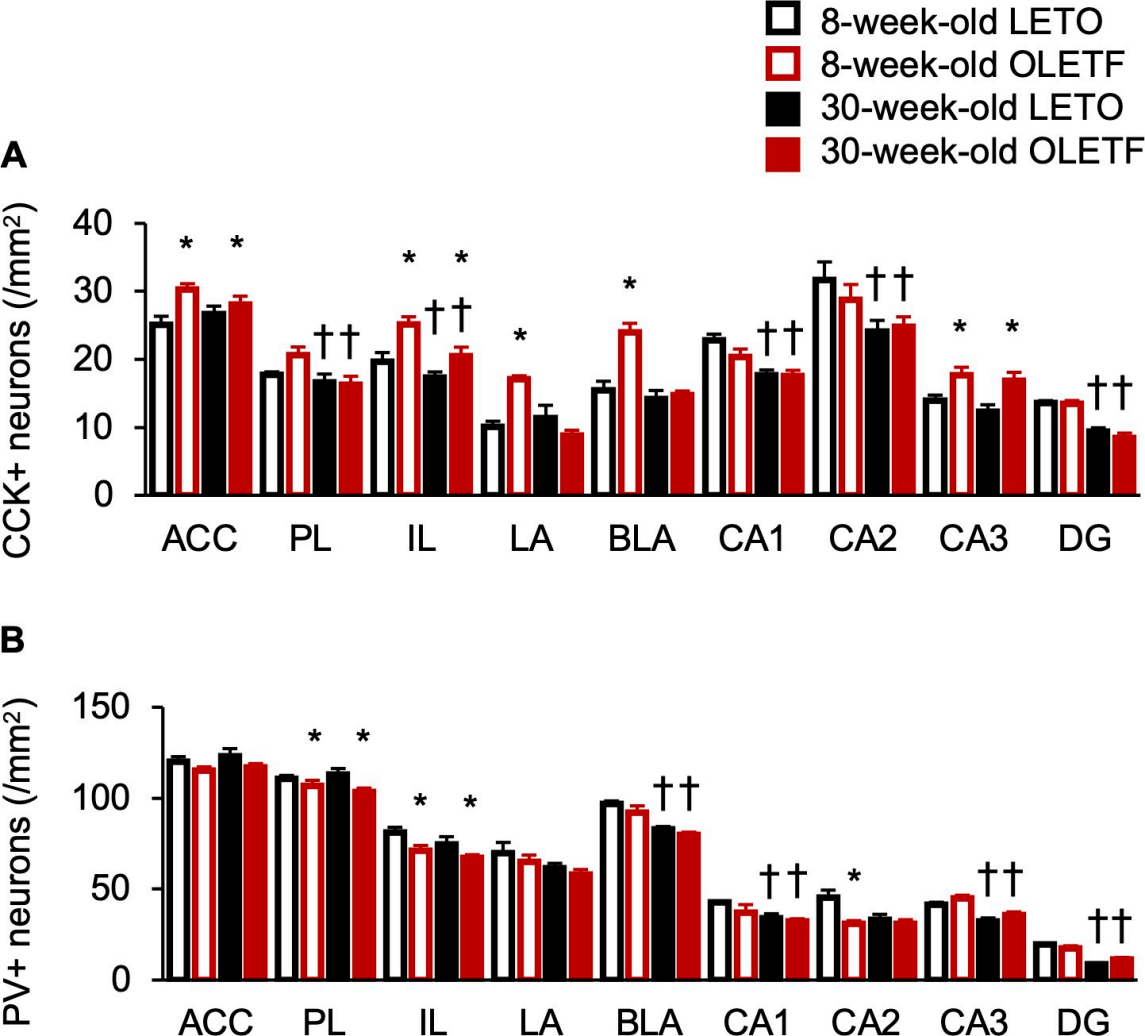
Densities of CCK- and PV-positive neurons in the corticolimbic regions. Densities of cholecystokinin-positive (CCK+; **A**) and parvalbumin-positive (PV+; **B**) neurons in the anterior cingulate cortex (ACC), prelimbic cortex (PL), infralimbic cortex (IL), lateral amygdala (LA), basolateral amygdala (BLA), hippocampal cornu ammonis (CA) area 1 (CA1), CA2, CA3, and dentate gyrus (DG). Values represent mean ± standard error of means. * Significant difference from age-matched LETO rats, *p* < 0.05; † significant difference from same strain at 8 weeks of age, *p* < 0.05, two-way ANOVA.

The densities of CCK-positive neurons of OLETF rats were significantly higher than those of LETO rats in the ACC, IL, and CA3 regardless of age (Fig 5A); there were significant main effects of strain (ACC: F[1, 18] = 8.59, *p* < 0.01; IL: F[1, 18] = 13.67, *p* < 0.01; CA3: F[1, 18] = 14.19, *p* < 0.01), but there were no significant interactions between strain and age. Meanwhile, the densities of CCK-positive neurons of OLETF rats were significantly higher than those of LETO rats in the LA and BLA only at 8 weeks old (Fig 5A); there were significant interactions between strain and age (LA: F[1, 18] = 14.85, *p* < 0.01; BLA: F[1, 18] = 12.55, *p* < 0.01) and a significant simple main effect at 8 weeks old (LA: F[1, 18] = 14.67, *p* < 0.01; BLA: F[1, 18] = 26.26, *p* < 0.01). Furthermore, the densities of CCK-positive neurons were significantly decreased with age in the PL, IL, CA1, CA2, and DG, regardless of strain (Fig 5A), and there were significant main effects of age (IL: F[1, 18] = 5.87, *p* < 0.05, F[1, 18] = 8.85, *p* < 0.01; CA1: F[1, 18] = 18.35, *p* < 0.01; CA2: F[1, 18] = 8.66, *p* < 0.01; DG: F[1, 18] = 63.37, *p* < 0.01).

The densities of PV-positive neurons in OLETF rats were significantly lower than those of LETO rats in the PL and IL, regardless of age (Fig 5B), and there were significant main effects of strain (PL: F[1, 18] = 6.14, *p* < 0.05; IL: F[1, 18] = 7.08, *p* < 0.05), but there were no significant interactions between strain and age. Meanwhile, the density of PV-positive neurons in OLETF rats was significantly lower than that of LETO rats in the CA2 only at 8 weeks old (Fig 5B). There was a significant interaction between strain and age (F[1, 18] = 4.76, *p* < 0.05) and a significant simple main effect of strain at 8 weeks old (F[1, 18] = 11.46, *p* < 0.01). Furthermore, the densities of PV-positive neurons decreased with age in the BLA, CA1, CA3, and DG regardless of strain (Fig 5B); there were significant main effects of age (BLA: F[1, 18] = 35.08, *p* < 0.01; CA1: F[1, 18] = 6.13, *p* < 0.05; CA3; F[1, 18] = 26.68, *p* < 0.01), a significant interaction between strain and age (DG: F[1, 18] = 5.73, *p* < 0.05), and significant simple main effects of age in both strains (DG in LETO rats: F[1, 18] = 59.48, *p* < 0.01; DG in OLETF rats: F[1, 18] = 18.72, *p* < 0.01). The densities of PV-positive neurons were comparable between strain and age in the ACC and LA (Fig 5B); there were no significant main effects of strain or age.

OLETF rats exhibited the opposite results with higher and lower densities of CCK- and PV-positive neurons in several corticolimbic regions; therefore, to investigate the relationships between CCK- and PV-positive neurons, we performed correlation analyses among the densities of these neurons in which the corticolimbic regions differed between strains. The density of CCK-positive neurons in the ACC was significantly and negatively correlated with the density of PV-positive neurons in the CA2 (ρ = -0.64, *p* < 0.01). We also carried out a correlation analysis for each primary antibody, CCK and PV, detected significant differences between strains. Statistical analyses indicated significant positive correlations among the densities as follows: CCK-positive neurons in the ACC and those in the IL (ρ = 0.66, *p* < 0.01); CCK-positive neurons in the IL and those in the BLA (ρ = 0.63, p < 0.01); CCK-positive neurons in the LA and those in the BLA (ρ = 0.64, *p* < 0.01); PV-positive neurons in the PL and those in the IL (ρ = 0.56, *p* < 0.01). In addition, the densities of CCK- and PV-positive neurons were not significantly correlated with the AUCs of glucose and insulin (S1 Table).

### Relationships between alterations of behavior and histological properties

To investigate the possible involvement of the histological alterations of OLETF rats in emotional behavior in the open-field test, we conducted correlation analyses between behavioral and histological parameters in both strains at both ages. Locomotion in the peripheral zone was significantly and negatively correlated with the density of CCK-positive neurons in the ACC (ρ = -0.58, *p* < 0.01) and positively correlated with the area of the mPFC (ρ = 0.65, *p* < 0.01). Latency to leave the center zone was significantly and negatively correlated with the density of CCK-positive neurons in the LA (ρ = -0.59, *p* < 0.01).

### Caspase-3 and ATF4 expressions in the corticolimbic system

To investigate whether the area reduction of the mPFC of OLETF rats, decreases in CCK- and PV-positive neurons with age regardless of strain, and decreases in PV-positive neurons of OLETF rats were due to apoptosis, we performed immunohistochemistry for caspase-3. However, no caspase-3-positive neurons were detected in the corticolimbic system of LETO and OLETF rats (S1 Fig).

**Fig 6:**
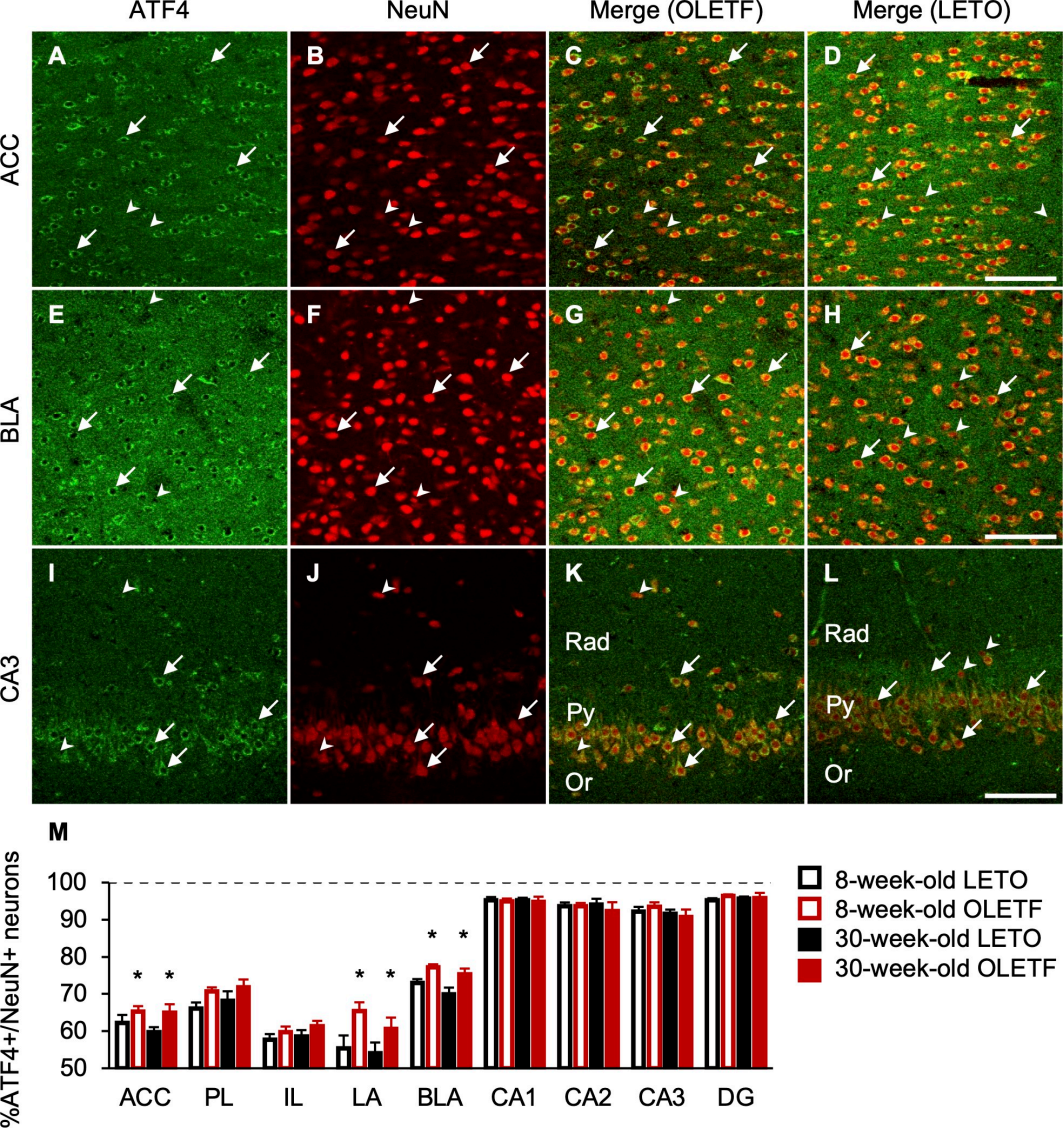
ATF4- and NeuN-positive neurons in the corticolimbic system. Representative immunofluorescent images of 8-week-old OLETF and LETO rats in the anterior cingulate cortex (ACC; **A**, **B**, **C** and **D**), basolateral amygdala (BLA; **E**, **F**, **G**, and **H**), and hippocampal cornu ammonis area 3 (CA3; **I**, **J**, **K**, and **L**. Labeling for activating transcription factor 4 (ATF4; **A**, **E**, and **I**) and neuronal nuclei (NeuN; **B**, **F**, and **G**) and merged images (**C**, **G**, and **K** for OLETF rats; **D**, **H**, and **L** for LETO rats). Arrows or arrow heads indicate neurons expressing ATF4 and NeuN or only NeuN, respectively. Scale bars = 100 μm. Rad: radiatum layer; Py: pyramidal cell layer; Or: oriens layer. The percentages of neurons co-expressing ATF4 and NeuN in the ACC, prelimbic cortex (PL), infralimbic cortex (IL), lateral amygdala (LA), BLA, CA1, CA2, CA3, and dentate gyrus (DG; **M**). ATF4+: ATF4-positive; NeuN+: NeuN-positive. Values represent mean ± standard error of means. * Significant difference from age-matched LETO rats, *p* < 0.05, two-way ANOVA.

**Fig 7:**
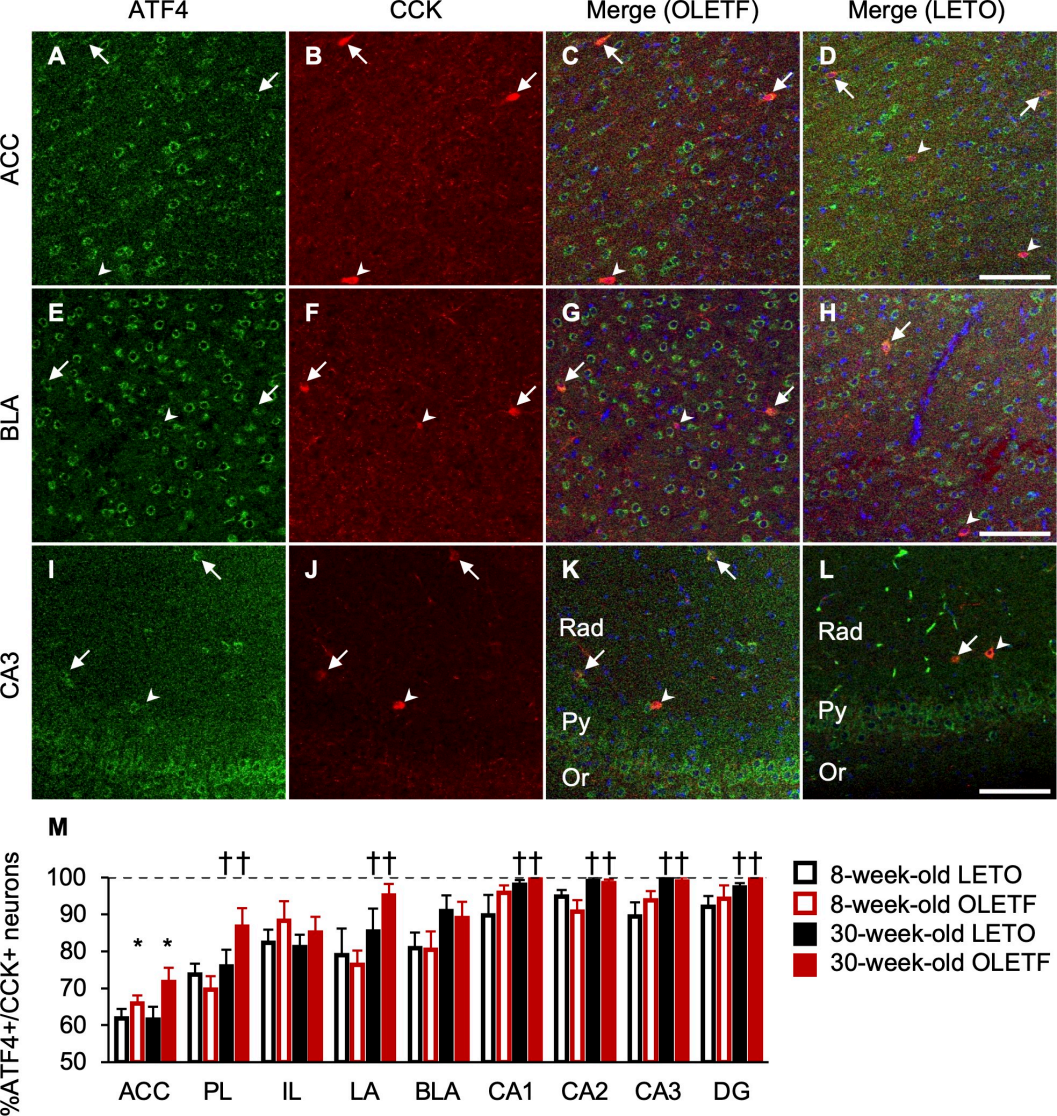
ATF4- and CCK-positive neurons in the corticolimbic system. Representative immunofluorescent images of 8-week-old OLETF and LETO rats in the anterior cingulate cortex (ACC; **A**, **B**, **C**, and **D**), basolateral amygdala (BLA; **E**, **F**, **G**, and **H**), and hippocampal cornu ammonis area 3 (CA3; **I**, **J**, **K**, and **L**). Labeling for activating transcription factor 4 (ATF4; **A**, **E**, and **I**) and cholecystokinin (CCK; **B**, **F**, and **J**) and merged images (**C**, **G**, and **K** for OLETF rats; **D**, **H**, and **L** for LETO rats). Arrows or arrow heads indicate neurons expressing ATF4 and CCK or only CCK, respectively. Scale bars = 100 μm. Rad: radiatum layer; Py: pyramidal cell layer; Or: oriens layer. The percentages of neurons co-expressing ATF4 and CCK in the ACC, prelimbic cortex (PL), infralimbic cortex (IL), lateral amygdala (LA), BLA, CA1, CA2, CA3, and dentate gyrus (DG; **M**). ATF4+: ATF4-positive; CCK+: CCK-positive. Values represent mean ± standard error of means. * Significant difference from age-matched LETO rats, *p* < 0.05; † significant difference from same strain at 8 weeks of age, *p* < 0.01, two-way ANOVA.

**Fig 8:**
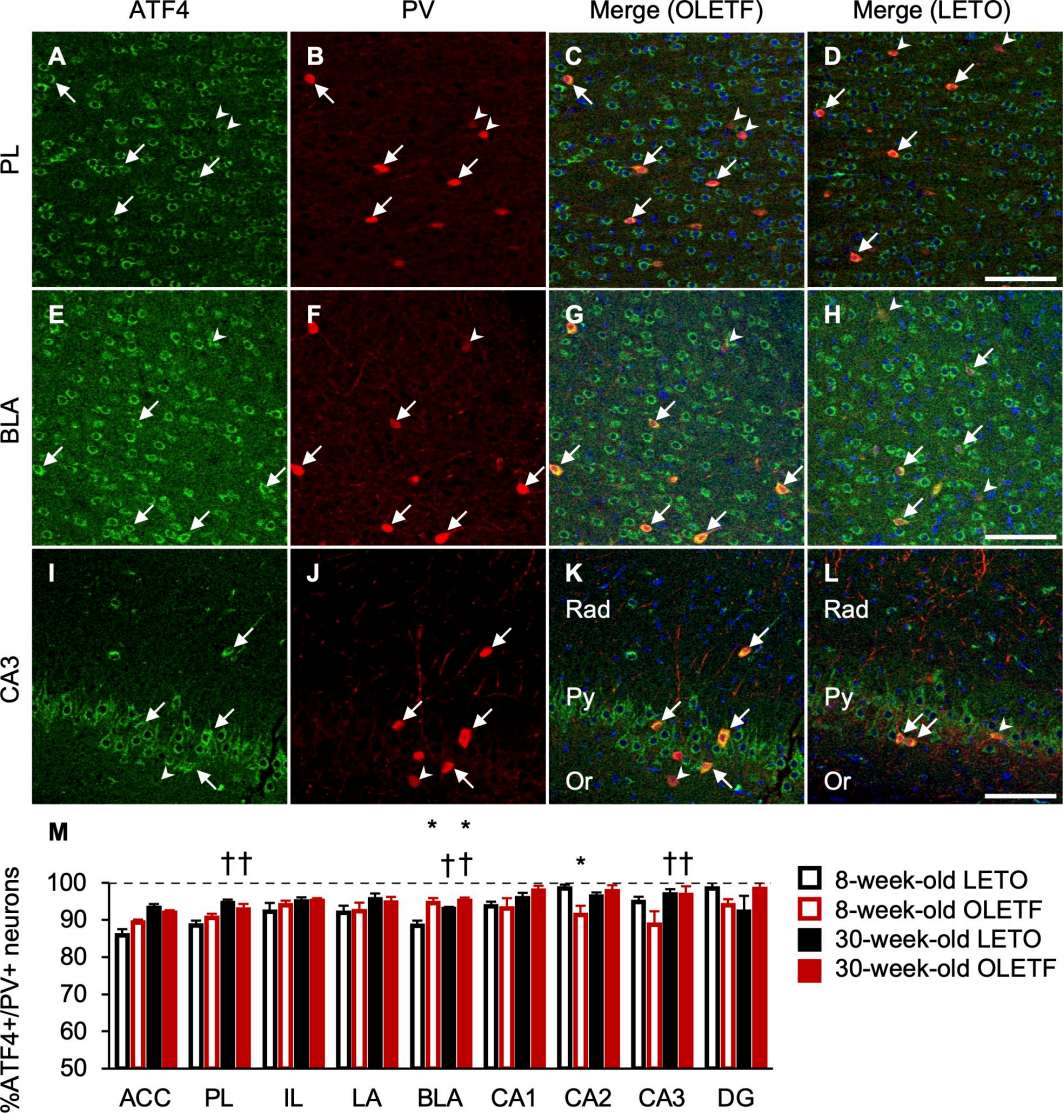
ATF4- and PV-positive neurons in the corticolimbic system. Representative immunofluorescent images of 8-week-old OLETF and LETO rats in the prelimbic cortex (PL; **A**, **B**, **C** and **D**), basolateral amygdala (BLA; **E**, **F**, **G**, and **H**), and hippocampal cornu ammonis area 3 (CA3; **I**, **J**, **K**, and **L**). Labeling for activating transcription factor 4 (ATF4; **A**, **E**, and **I**) and parvalbumin (PV; **B**, **F**, and **J**) and merged images (**C**, **G**, and **K** for OLETF rats; **D**, **H**, and **L** for LETO rats). Arrows or arrow heads indicate neurons expressing ATF4 and PV or only PV, respectively. Scale bars = 100 μm. Rad: radiatum layer; Py: pyramidal cell layer; Or: oriens layer. The percentages of neurons co-expressing ATF4 and PV in the anterior cingulate cortex (ACC), PL, infralimbic cortex (IL), lateral amygdala (LA), BLA, CA1, CA2, CA3, and dentate gyrus (DG; **M**). ATF4+: ATF4-positive; PV+: PV-positive. Values represent mean ± standard error of means. * Significant difference from age-matched LETO rats, *p* < 0.01; † significant difference from same strain at 8 weeks of age, *p* < 0.05, two-way ANOVA.

We also performed immunofluorescence for ATF4/NeuN, CCK, and PV (Figs 6, 7, and 8). ATF4 was mainly localized in the cytoplasm in the mPFC, BLC, and hippocampus (Fig 6). The percentages of neurons co-expressing ATF4 and NeuN in OLETF rats were significantly higher than those of LETO rats in the ACC, LA, and BLA, regardless of age (Fig 6M), and there was a significant main effect of strain (ACC: F[1, 18] = 4.57, *p* < 0.05; LA: F[1, 18] = 7.62, *p* < 0.05; BLA: F[1, 18] = 10.57, *p* < 0.01), but there were no significant interactions between strain and age. The percentages of neurons co-expressing ATF4 and NeuN were comparable between strain and age in the PL, IL, CA1, CA2, CA3, and DG (Fig 6M); there were no significant main effects of strain and age. There were hardly any cells expressing only ATF4 in the mPFC, BLC, and hippocampus. Based on the observations of double staining for ATF4 with CCK or PV, ATF4 co-expression was more frequent in PV-positive neurons than in CCK-positive neurons in the mPFC and BLC. In line with the co-expression of ATF4 and NeuN, the percentage of neurons co-expressing ATF4 and CCK in OLETF rats was significantly higher than that of LETO rats in the ACC regardless of age (Fig 7M), and there was a significant main effect of strain (F[1, 18] = 4.80, *p* < 0.05), but there was no significant interaction between strain and age. Furthermore, the percentages of neurons co-expressing ATF4 and CCK increased with age in the PL, LA, CA1, CA2, CA3, and DG, regardless of strain (Fig 7M), and there were significant main effects of age (PL: F[1, 18] = 4.97, *p* < 0.05; LA: F[1, 18] = 5.56, *p* < 0.05; CA1: F[1, 18] = 5.21, *p* < 0.05; CA2: F[1, 18] = 11.13, *p* < 0.01; CA3: F [1, 18] = 13.54, *p* < 0.01; DG: F[1, 18] = 6.16, *p* < 0.05). The percentages of neurons co-expressing ATF4 and CCK were comparable between strain and age in the IL and BLA (Fig 7M); there were no significant main effects of strain and age. In addition, in line with the co-expression of ATF4 and NeuN, the percentage of neurons co-expressing ATF4 and PV in OLETF rats was significantly higher than that of LETO rats in the BLA regardless of age (Fig 8M), and there was a significant main effect of strain (F[1, 18] = 14.46, *p* < 0.01), but there was no significant interaction between strain and age. The percentage of neurons co-expressing ATF4 and PV of OLETF rats also tended to be higher than that of LETO rats in the ACC only at 8 weeks old (Fig 8M). There was a significant interaction between strain and age (F[1, 18] = 4.52, *p* < 0.05) and a simple main effect of strain at 8 weeks old (F[1, 18] = 4.32, *p* = 0.05). Meanwhile, the percentage of neurons co-expressing ATF4 and PV of OLETF rats was significantly lower than that of LETO rats in the CA2 only at 8 weeks old (Fig 8M); there was a significant interaction between strain and age (F[1, 18] = 6.68, *p* < 0.05) and a simple main effect of strain at 8 weeks old (F[1, 18] = 8.48, *p* < 0.01). Furthermore, the percentages of neurons co-expressing ATF4 and PV increased with age in the PL, BLA, and CA3 regardless of strain (Fig 8M); there were significant main effects of age (PL: F[1, 18] = 10.67, *p* < 0.01; BLA: F[1, 18] = 5.50, *p* < 0.05; CA3: F[1, 18] = 4.58, *p* < 0.05). The percentages of neurons co-expressing ATF4 and PV were comparable between strain and age in the IL, LA, CA1, and DG (Fig 8M); there were no significant main effects of strain and age.

## Discussion

Our results indicate that OLETF rats show altered emotionality, reduction in the area of the mPFC, and increased CCK- and decreased PV-positive neurons in the corticolimbic system at the prediabetic stage. First, in the open-field test, the locomotion in the center zone was lower and the latency to leave the center zone was longer in OLETF rats at 8 and 30 weeks old, respectively, indicating altered emotional behavior. Second, the brain weight and regional area of the mPFC were smaller in OLETF rats at both 8 and 30 weeks old. Third, the densities of CCK- and PV-positive neurons in several corticolimbic regions were higher and lower in OLETF rats at both 8 and 30 weeks old. Finally, no apoptosis was detected by immunohistochemistry for caspase-3 regardless of strain and age, whereas the percentages of CCK- and PV-positive neurons co-expressing ATF4 in the ACC and BLA were higher in OLETF rats at both 8 and 30 weeks old.

The first interest of the present study was the time when the previous results observed in 20-week-old OLETF rats emerged. In the present study, 8-week-old OLETF rats at the prediabetic stage already showed area reduction of the mPFC and increased CCK- and decreased PV-positive neurons in the corticolimbic system. However, taking into consideration the temporal changes of CCK- and PV-positivity in our previous study [11], OLETF rats showed more CCK-positive neurons in the amygdala at 8 and 20 weeks old but not at 30 weeks old and fewer PV-positive neurons in the mPFC and hippocampus at 8 and 30 weeks old but not at 20 weeks old. Biochemical profiles vary by stage in diabetes; indeed, OLETF rats showed progressive hyperglycemia from 8 weeks old and hyperinsulinemia only at 20 weeks old in our studies [10,11]. Intraperitoneal insulin injections reportedly had a neuroprotective effect by suppressing glutamate levels in the plasma and cerebrospinal fluid, not by suppression of plasma glucose levels as in high fat diet-induced diabetic rats [46]. Although temporal changes of glutamate levels have not been examined in OLETF rats, it is possible that changing insulin levels might be associated with decreasing numbers of PV-positive neurons in OLETF rats. In addition, streptozotocin-induced diabetic rats reportedly showed increased corticotropin-releasing factor (CRF) and CRF-1 receptor mRNA levels in the hypothalamus [47]. Repeated stimulation of the CRF-1 receptor by cortagine reportedly increased CCK mRNA level in the LA and BLA [25]. Although temporal changes of CRF and CRF-1 receptor expressions have not been examined in OLETF rats, these results indicate that altered CRF-1 receptor expression might be involved in the increase in CCK-positive neurons in the amygdala of OLETF rats. CCK reportedly increases anxiety-like behavior through the activation of the CCK-2 receptor [48], particularly in the amygdala [49]. The increased CCK-positive neurons in the amygdala at the prediabetic and early stage of type 2 diabetes in OLETF rats might be involved in specific anxiety-related phenotypes in the open field test found at the stages, such as decreased locomotion in the center zone. As for emotional behaviors, consistent with previous studies using 9- to 22-week-old OLETF rats [11–14,50], OLETF rats exhibited hypolocomotion regardless of age and anxiety-related behaviors, as indicated by decreased locomotion in the center zone and increased grooming at 8 weeks old [11,14,51] and increased latency to leave the center zone at 30 weeks old [14] in the open-field test. OLETF rats showed lower velocities of locomotive behaviors in each zone regardless of age, but the velocity in the center zone until first escape was comparable at 30 weeks old; therefore, increased latency to leave the center zone could not simply due to hypolocomotion. However, we cannot clearly conclude that OLETF rats showed increased anxiety-like behavior in the present study because the time spent in the center zone after re-entering this area was comparable between LETO and OLETF rats. Further studies are needed to examine anxiety-like behavior comprehensively using other behavioral tests such as the elevated plus maze test. Locomotion in the total and peripheral zones was decreased in 8- and 30-week-old OLETF rats as compared to LETO rats in the present study, but not in 20-week-old OLETF rats in our previous study [11], although the number of rearing was fewer in 8-, 20-, and 30-week-old OLETF rats. Intraperitoneal insulin injections reportedly recovered the decreased number of squares crossing and rearing in a hole board test in streptozotocin-induced diabetic mice without complete recovery of blood glucose levels [52]. Plasma insulin level was higher in OLETF rats at only 20 weeks old [10,11]. These results indicate that plasma insulin levels might be involved in the differences of locomotion in the open-field test among different ages. It is noteworthy that these behavioral and histological alterations in 8-week-old OLETF rats were not further exacerbated in 30-week-old OLETF rats. Kobayashi et al. [50] also reported that locomotor activity in the open-field test was comparable between 9- and 22-week-old OLETF rats, although they did not reveal any significant changes in neural mechanisms. The present study provides further evidence that OLETF rats at the prediabetic stage already exhibit altered emotional behavior and associated alterations in their neural mechanisms. It is possible that these behavioral and histological alterations may already reach plateaus at the prediabetic stage in OLETF rats; otherwise, these rats have an innate characteristic. In contrast, 13-months-old GK rats, exhibiting more severe fasting hyperglycemia than 30-week-old OLETF rats, reportedly showed type 2 diabetes progression-dependent decreases in NeuN-positive cells in the cerebral cortex, glutamic acid decarboxylase-67-positive cells in the striatum, and calbindin-positive cells in the cerebral cortex and striatum [40,53]. Therefore, further studies should be carried out by time points at the late severe stage of type 2 diabetes in OLETF rats; nevertheless, we found no significant associations between metabolic characteristics and histological parameters in the present study. In addition, the onset of hyperglycemia may be involved in the behavioral and histological alterations of OLETF rats because 8-week-old OLETF rats already exhibited higher blood glucose levels than age-matched LETO rats. We are now beginning to determine whether the onset of hyperglycemia affects these alterations in OLETF rats placed on food restriction. Further studies are needed to clarify the emerging time point of significant differences in OLETF rats and the involvement in diabetes.

To investigate the pathological processes underlying the reduction of the mPFC area of OLETF rats, decreases in PV-positive neurons of OLETF rats, and decreases in CCK- and PV-positive neurons in the corticolimbic system with age regardless of strain, we performed immunohistochemistry for caspase-3, which indicates induction of apoptosis. Diabetes can induce apoptosis in the brain, as demonstrated by increased caspase-3 expression [36], and hypoxia–ischemia-induced brain volume shrinkage is reportedly associated with neuronal apoptosis demonstrated by increased caspase-3 expression [54]. However, caspase-3-positive cells were not detected in the mPFC, BLC, or hippocampus of LETO and OLETF rats at either age, but caspase-3-positive cells were confirmed in a positive control sample from a rat model of traumatic brain injury using the same immunohistochemical method. Therefore, induction of apoptosis could not be demonstrated in area reductions of the mPFC of OLETF rats, decreases in PV-positive neurons of OLETF rats, and decreases in CCK- and PV-positive neurons with age. In contrast, ATF4 co-expression was specifically increased in OLETF rats in NeuN-, CCK-, and PV-positive neurons in the ACC, LA, and BLA. In addition, ATF4 co-expression in the corticolimbic system increased globally with age, regardless of strain. Previous studies also showed that GK rats exhibited increased ATF4 expression in PV-positive neurons in the hippocampus but not induction of apoptosis, as demonstrated by caspase-3 and CHOP expression and TUNEL staining [39,40], and ATF4 expression increased with age in whole brain lysates [55]. ATF4 reportedly induced specific reductions of cholinergic neurons more than global neuronal losses detected by TUNEL staining in the forebrain, indicating that ATF4 could induce cell type-specific reduction without inducing neuronal apoptosis [41]. In the present study, the co-expression patterns of ATF4 between the groups did not completely match the patterns in the number of CCK- and PV-positive neurons. It is possible that increased ATF4 expression without induction of apoptosis in the brain is one of the pathological features of type 2 diabetes already found at the prediabetic stage.

Diabetic GK rats reportedly showed increased PV-positive neurons in the hippocampus at 6 months old [39]; meanwhile, 3- and 13-month-old GK rats showed PV-positive neurons in the cortex and striatum that were comparable with those of control Wistar rats [40]. In contrast, in the present study, we found increased CCK- and decreased PV-positive neurons in the mPFC, BLA, and hippocampus in OLETF rats at 8 weeks old, consistent with our previous study in 20-week-old OLETF rats [11]. In addition, 6-month-old GK rats showed increased ATF4 expression in PV-positive neurons in the hippocampus [39]. In contrast, 8-week-old OLETF rats exhibited decreased ATF4 expression in PV-positive neurons in the CA2, whereas ATF4 co-expression in PV-positive neurons in the hippocampus was comparable between LETO and OLETF rats at 30 weeks old. The discrepancies between OLETF and GK rats may be due to the different pathological properties of type 2 diabetes: chronic progression of type 2 diabetes with obesity and temporal alterations of plasma insulin levels in OLETF rats; continuous hypoinsulinemia and non-obesity in GK rats [53]; however, the causal mechanisms of alterations of CCK- and PV-positive neurons and ATF4 co-expression in OLETF rats are still unclear. Correlation analysis revealed that a typical trend of results through the groups for CCK- and PV-positive neurons was observed along with increased CCK-positive and decreased PV-positive neurons in OLETF rats. These data suggest that neurological changes have already occurred not focally but broadly in the corticolimbic system at the prediabetic stage, at 8 weeks old.

## Conclusion

We demonstrated that OLETF rats at the prediabetic stage already exhibited altered emotionality, mPFC area reduction, and increased CCK-positive and decreased PV-positive neurons in the corticolimbic system. In addition, OLETF rats showed increased ATF4 expression in the corticolimbic system without induction of apoptosis at the prediabetic stage. To our knowledge, this is the first study to investigate anxiety-like behavior and associated neural mechanisms at different pathological stages in a model of type 2 diabetes. Although this study is limited to analyses of the relationships between altered emotional behaviors, regional area reduction of the mPFC, and altered numbers of CCK and PV in the corticolimbic system in OLETF rats, the possible mechanisms underlying changes in emotions such as anxiety in type 2 diabetes may be valuable for the treatment and prevention of the prediabetic stage.

## Supporting information

S1 Fig Immunohistochemistry for caspase-3. Representative images of the somatosensory cortex of a traumatic brain injury model rat without (**A**) or with (**B**) primary antibody. Arrows indicate caspase-3-positive cells. Representative images of 8-week-old LETO (**C**, **G**, **K**), 8-week-old OLETF (**D**, **H**, **L**), 30-week-old LETO (**E**, **I**, **M**), and 30-week-old OLETF (**F**, **J**, **N**) rats in the anterior cingulate cortex (ACC; **C**, **D**, **E**, **F**), basolateral amygdala (BLA; **G**, **H**, **I**, **J**), and hippocampal cornu ammonis area 3 (CA3; **K**, **L**, **M**, **N**). Scale bars = 100 μm. Rad: radiatum layer; Py: pyramidal cell layer; Or: oriens layer.
(TIFF)

S1 Table Relationships between glucose and insulin levels and CCK- and PV-positive neurons. Values represent Spearmann’s rank correlation coefficients. All relationships were not significant. AUC: area under the curve; CCK: cholecystokinin; PV: parvalbumin; ACC: anterior cingulate cortex; IL: infralimbic cortex; LA: lateral amygdala; BLA: basolateral amygdala; CA3: hippocampal cornu ammonis area 3; PL: prelimbic cortex; CA2: hippocampal cornu ammois area 2.
(DOCX)
