# Supplementary figures and images for: Medial prefrontal area reductions, altered expressions of cholecystokinin, parvalbumin, and activating transcription factor 4 in the corticolimbic system, and altered emotional behavior in a progressive rat model of type 2 diabetes
Source: PLoS One. 2021 Sep 10;16(9):e0256655. doi: 10.1371/journal.pone.0256655 (PMC8432800; doi:10.1371/journal.pone.0256655)

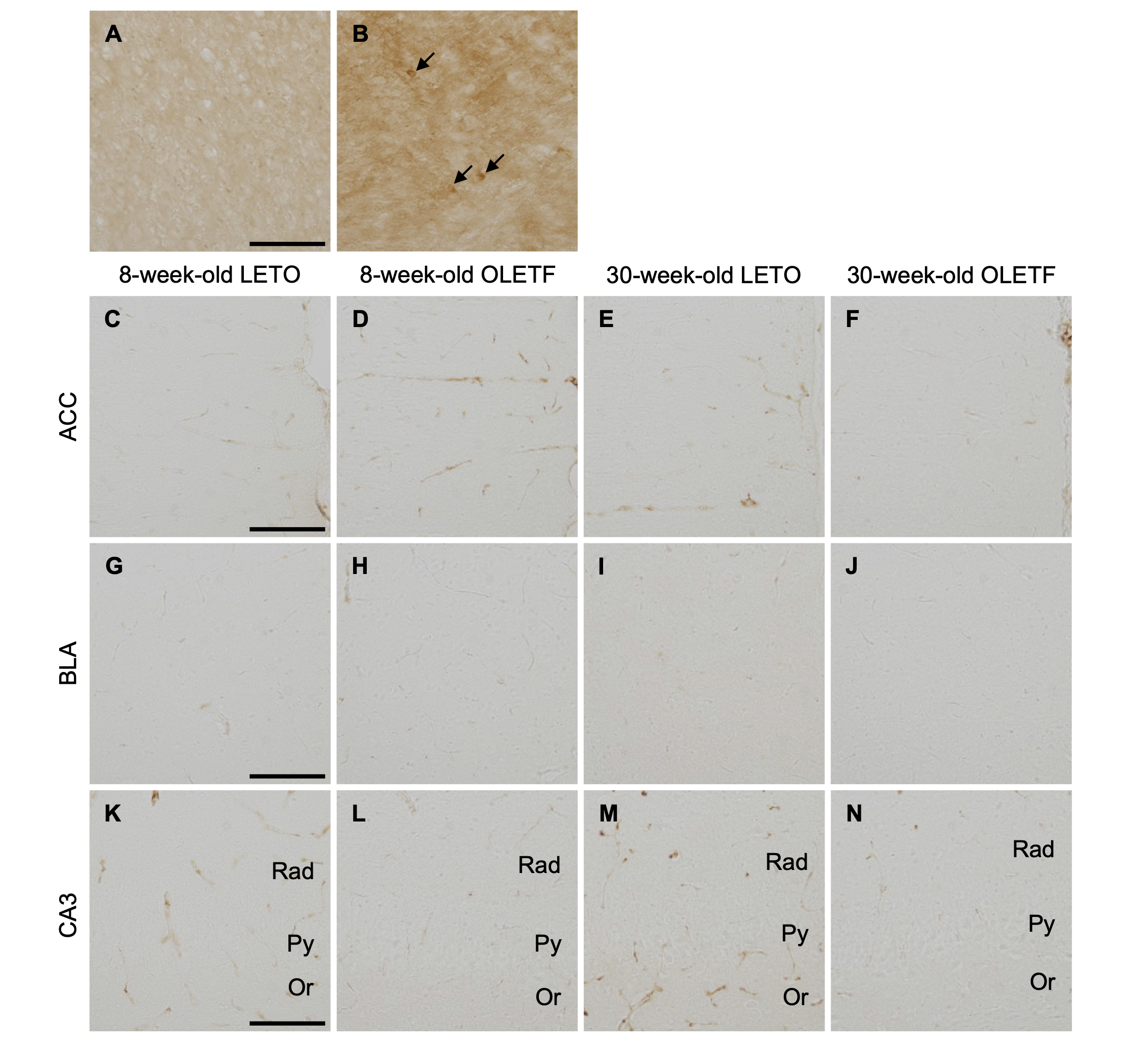

Supplement: S1 Fig — Representative images of the somatosensory cortex of a traumatic brain injury model rat without (A) or with (B) primary antibody. Arrows indicate caspase-3-positive cells. Representative images of 8-week-old LETO (C, G, K), 8-week-old OLETF (D, H, L), 30-week-old LETO (E, I, M), and 30-week-old OLETF (F, J, N) rats in the anterior cingulate cortex (ACC; C, D, E, F), basolateral amygdala (BLA; G, H, I, J), and hippocampal cornu ammonis area 3 (CA3; K, L, M, N). Scale bars = 100 μm. Rad: radiatum layer; Py: pyramidal cell layer; Or: oriens layer. (TIFF) [file pone.0256655.s001.tiff]
