# Supplementary material for: Medial prefrontal area reductions, altered expressions of cholecystokinin, parvalbumin, and activating transcription factor 4 in the corticolimbic system, and altered emotional behavior in a progressive rat model of type 2 diabetes
Source: PLoS One. 2021 Sep 10;16(9):e0256655. doi: 10.1371/journal.pone.0256655 (PMC8432800; doi:10.1371/journal.pone.0256655)
Supplement: S1 Table — Values represent Spearmann’s rank correlation coefficients. All relationships were not significant. AUC: area under the curve; CCK: cholecystokinin; PV: parvalbumin; ACC: anterior cingulate cortex; IL: infralimbic cortex; LA: lateral amygdala; BLA: basolateral amygdala; CA3: hippocampal cornu ammonis area 3; PL: prelimbic cortex; CA2: hippocampal cornu ammois area 2. (DOCX) [file pone.0256655.s002.docx]

**S1 Table. Relationships between glucose and insulin levels and CCK- and PV-positive neurons.**

|  |  | Glucose AUC | Insulin AUC |
| --- | --- | --- | --- |
| CCK-positive neurons | ACC | ρ = 0.43 | ρ = 0.05 |
|  | IL | ρ = 0.16 | ρ = − 0.21 |
|  | LA | ρ = − 0.05 | ρ = − 0.32 |
|  | BLA | ρ = 0.08 | ρ = − 0.45 |
|  | CA3 | ρ = 0.41 | ρ = 0.02 |
| PV-positive neurons | PL | ρ = − 0.30 | ρ = − 0.14 |
|  | IL | ρ = − 0.42 | ρ = − 0.27 |
|  | CA2 | ρ = − 0.46 | ρ = − 0.14 |

Values represent Spearmann’s rank correlation coefficients. All relationships were not significant. AUC: area under the curve; CCK: cholecystokinin; PV: parvalbumin; ACC: anterior cingulate cortex; IL: infralimbic cortex; LA: lateral amygdala; BLA: basolateral amygdala; CA3: hippocampal cornu ammonis area 3; PL: prelimbic cortex; CA2: hippocampal cornu ammois area 2.
